# Quantitative determination of FAM3D plasma protein in type 2 diabetes mellitus

**DOI:** 10.1186/1758-5996-7-S1-A137

**Published:** 2015-11-11

**Authors:** José Antônio Januário Neves, Daniel Giannella Neto

**Affiliations:** 1 Universidade Nove de Julho, Manhuaçu, Brazil

## Background

Among the new cytokine Family with Sequence Similarity 3 (FAM3), a subfamily of proteins similar to cytokines known as FAM3D was identified. FAM3D is predominantly expressed in the gastrointestinal tract of normal individuals. Plasma concentrations of FAM3D range according to the nutritional status presenting a postprandial increase and a reduction in the later post-absorptive period. It might be speculated that FAM3D is an inhibitor of insulin secretion, as it has been observed a significant increase in patients with pancreatic adenocarcinoma associated with T2D.

## Aims

Quantitatively determine the plasma concentration of FAM3D, in a group of patients with T2D and comparing with patients with pre-Type 2 Diabetes Mellitus (pT2D) and Control (CTRL) individuals paired according to gender, age and body mass index in order to better elucidate the physiology of FAM3D as well as its pathophysiological role in T2D.

## Materials and methods

We selected 90 patients comprising 15 male and 14 females diagnosed with T2D; 12 male and 15 female diagnosed pT2D; and 7 male and 9 female CTRL. Seventeen patients were excluded according to the inclusion and exclusion criteria. In total, 73 patients were included. All were submitted to measurement of anthropometric and biochemical tests, including HOMA-IR calculation and the determination of plasma concentrations of FAM3D by ELISA. The Mann-Whitney tests were used in the comparison of study groups, and the regression coefficient P of Spearman was calculated on correlation between plasma concentrations of FAM3D and other anthropometric and biochemical variables. The level of statistical significance was set at p <0.05.

## Results

Among the groups there were no significant difference in the concentration of FAM3D, and the median±Q for the CTRL group (24.92ng/mL ±9.11); pT2D (14.65ng/mL ±6.02) and T2D (19.87ng/mL ±5.39). The group of DM2 patients on sulfonylurea (23.42ng/mL ±6.1) had higher values compared to the group treated with other oral antidiabetic drugs (14.9 ng/mL±7.30), p <0.05.

## Conclusions

There was no correlation of FAM3D between groups. Patients with T2D on sulfonylurea presented a higher concentration of FAM3D when compared with T2D patients treated with other oral antidiabetic drugs. Although further studies are necessary, the increased concentrations of FMA3D in patients with T2D on sulfonylurea could be due to the inhibitory effect of the secretagogue on FAM3D.

